# Sodium metabisulfite–induced polymerization of sickle cell hemoglobin incubated in the extracts of three medicinal plants (*Anacardium occidentale, Psidium guajava,* and *Terminalia catappa*)

**DOI:** 10.4103/0973-1296.80670

**Published:** 2011

**Authors:** Paul Chidoka Chikezie

**Affiliations:** *Department of Biochemistry, Imo State University, Owerri, Imo State, Nigeria*

**Keywords:** *A. occidentale*, DeoxyHbS, Polymerization, *P. guajava*, *T. catappa*

## Abstract

**Background:**

The exploitation and utilization of vast varieties of herbal extracts may serve as alternative measures to deter aggregation of deoxygenated sickle cell hemoglobin (deoxyHbS) molecules.

**Objective:**

The present *in vitro* study ascertained the capacity of three medicinal plants, namely, *Anacardium occidentale, Psidium guajava*, and *Terminalia catappa*, to alter polymerization of HbS.

**Materials and Methods:**

Spectrophotometric method was used to monitor the level of polymerization of hemolysate HbS molecules treated with sodium metabisulfite (Na_2_ S_2_ O_5_) at a regular interval of 30 s for a period of 180 s in the presence of separate aqueous extracts of *A. occidentale, P. guajava*, and *T. catappa*. At time intervals of 30 s, the level of polymerization was expressed as percentage of absorbance relative to the control sample at the 180th s.

**Results:**

Although extracts of the three medicinal plants caused significant (*P* < 0.05) reduction in polymerization of deoxyHbS molecules, the corresponding capacity in this regard diminished with increase in incubation time. Aqueous extract of *P. guajava* exhibited the highest capacity to reduced polymerization of deoxyHbS molecules. Whereas at *t* > 60 s, extract concentration of 400 mg% of *A. occidentale* activated polymerization of deoxyHbS molecules by 6.23±1.34, 14.53±1.67, 21.15±1.89, and 24.42±1.09%, 800 mg% of *T. catappa* at *t* > 30 s gave values of 2.50±1.93, 5.09±1.96, 10.00±0.99, 15.38±1.33, and 17.31±0.97%.

**Conclusion:**

The capacity of the three medicinal plants to interfere with polymerization of deoxyHbS molecules depended on the duration of incubation and concentration of the extracts.

## INTRODUCTION

Cashew (*Anacardium occidentale*) is a multipurpose tree of the Amazon and African rainforest that grows up to 15 m high. It has a thick and tortuous trunk with branches so winding that they frequently reach the ground. The cashew fruit is a rich source of vitamins, minerals, and other essential nutrients.[1] Volatile compounds present in the fruit include esters, terpenes, and carboxylic acids.[2] The bark and leaves of cashew are a rich source of tannins, a group of plant chemicals with documented biological activity.[3] The bark, leaves, and shell oil around the nut are used medicinally as anti-inflammatory[3] and astringent preparations, which may be why cashew is effective in treating diarrhea.[4] Several clinical studies have shown that these biochemicals curb the darkening effect of aging by inhibiting tyrosinase activity,[5] protective against laboratory-induced diabetes,[6,7] treatment of leishmanial ulcers due to *Leishmania* (Viannia)[8] and are toxic to certain cancer cells.[1] Antimicrobial properties of *A. occidentale* against *E. coli*,[4,9] *Pseudomonas*,[4] and *Helicobacter pylori* considered to cause acute gastritis and stomach ulcers have been reported.[10]

Guava (*Psidium guajava*) is a tropical and semi-tropical plant. The branches are crooked, causing opposite leaves to overlap. The flowers are white, incurved petals, 2 or 3 in the leaf axils. The fruit is small, 3–6 cm long, pear-shaped, reddish-yellow when ripe.[11] Lectin chemicals in leaves of *P. guajava* have been shown to bind to *E. coli*,[12] preventing its adhesion to the intestinal wall and thus preventing infection.[13] *P. guajava* fruit intake decreases blood pressure and serum high-density lipoprotein/cholesterol levels.[14,15] The leaves of the guava tree in decoction are recommended for gastroenteritis,[16] ulcers, vaginal and uterine problems, and where an astringent remedy is needed.[1] Also, it has been used for spasms,[17] fevers, worm infections, kidney dysfunctions, epilepsy, diabetes, and cerebral infections.[1]

Indian almond (*Terminalia catappa*) tree can reach a height of 35 m (110 ft). It grows upright and forms a symmetrical crown with horizontal branches distinctively arranged in tiers. As the tree ages, the crown will become increasingly flattened and eventually take on a vase shape.[18] Indian almond leaves contain several different flavonoids, including kaempferol and quercetin.[19] Flavonoids have been shown to possess a range of biological activities that are consistent with them contributing to the protection against degenerative diseases, such as cancer, diabetes, cardiovascular diseases, and cataract.[20–22] It is also rich in various tannins; astringent, bitter plant polyphenols that either bind and precipitate or denature protein molecules.[19] While the juice of the leaves is used as a folk remedy against various ailments that damage the skin, such as scabies and leprosy, the leaves themselves are used to dress rheumatic joints.[23] The young leaves are ingested by people suffering from intestinal parasites, dysentery, and are believed to help get rid of colic in babies.[24]

The sickle cell hemoglobin (HbS) is a product of a defective genetic code of hemoglobin molecule.[25] The HbS molecule is prone to deoxygenation-induced polymerization and exhibits a relatively low insolubility. The reason for this phenomenon is the consequence of substitution on the beta (β) chain of HbS molecule, a polar glutamic acid residue with nonpolar valine molecule, thereby generating a sticky patch.[26,27] Specifically, only this one β^6Glu→Val^ residue of each tetramer resides in an intermolecular contact region for deoxyHbS aggregation. The hydrophobic valine side chain appears to fit into a hydrophobic pocket formed by β88 leucine and β85 phenylalanine residues on an adjacent hemoglobin molecule.[28] The normal glutamic acid would not easily fit into this pocket explaining at least part of why deoxyHbS does not polymerize. The model described in sequential steps includes nucleation, growth, and subsequent alignment of the molecule into microfibrils parallel to each other with the resultant membrane deformity and damage.[29,30] Characteristically, the red cells’ normal biconcave disc appearance is distorted to a spiculated sickle shape. This aberrant cell distortion accounts for the pathophysiology in this disease, namely, hemolytic anemia, vascular stasis, occlusion, and thrombosis.

The development of chemical modification agents that reduce the tendency of deoxyHbS to aggregate representsan important chemotherapeutic goal. Whereas hemoglobin carbamylation by cyanate[31,32] is a potentially effective antisickling agent, methyl acetylphosphate (MAP)has been reported to bind to the 2,3-diphosphoglycerate (2,3-DPG)binding site of hemoglobin, where it selectively acetylates residues,resulting in increased solubility of deoxyHbS molecules.[33] *In vitro* studies by Abdulmalik *et al*[34] reported that 5-hydroxymethyl-2-furfural (5HMF) forms a high-affinity Schiff-base adduct with HbS molecules and inhibits red cell sickling. Hydroxyurea[35] and 2-imidazolines[36] are among the few developed antisickling agents, which interfere and disrupt the contact point that promotes aggregation of deoxyHbS molecules.

However, limitations of these antisickling agents are undesirable structural andfunctional changes of hemoglobin as well as toxicity resulting from modifications of other protein molecules.[25,31–33] The exploitation and utilization of vast varieties of herbal extracts may serve as alternative measures to deter aggregation of deoxyHbS molecules. Moreover, the specimens are commonly consumed plant materials and pose little or no toxic effects. Therefore, the present study seeks to ascertain the capacity of three medicinal plants (*A. occidentale*, *P. guajava*, and *T. catappa*) to interfere with polymerization of HbS molecules.

## MATERIALS AND METHODS

### Collection of plant specimens

Fresh samples of *A. occidentale*, *P. guajava*, and *T. catappa* leaves were harvested between July and August 2010, from trees within the environment of Imo State University, Owerri, Nigeria. The plant specimens were identified and authenticated by Dr. F. N. Mbagwu at the Herbarium in the Department of Plant Science and Biotechnology. A voucher specimen was deposited at the Herbarium for reference purposes.

### Preparation of aqueous extract of plant specimen

The samples were washed under continuous current of distilled water for 15 min and air dried at room temperature for 60 min. The separate leaves were dried for 5 h in an oven at 60°C to become crispy, and ground with ceramic mortar and pestle. Two grams (2 g) each, of the pulverized specimen was suspended in 100 mL of distilled water and allowed to stand for 6 h at 37°C. The aqueous extracts (2 g%) of *A. occidentale*, *P. guajava*, and *T. catappa* leaves were obtained by filteration with Whatman No. 2 filter paper. The prepared extracts were kept at 4°C in a refrigerator for at least 24 h before subsequent tests. Serial dilutions of the aqueous extracts in the order of 200, 400, 600, and 800 mg% were used for polymerization analyses.

### Collection of blood samples/preparation of erythrocyte hemolysate

Five milliliters (5.0 mL) of human venous blood samples of HbSS genotype were collected by venipuncture and stored in EDTA anticoagulant tubes. The blood samples were obtained between July and August 2010, from 9 male volunteers (59-79 kg) in the age group of 21–34 years attending clinics at the Federal Medical Center (FMC), Imo State University Teaching Hospital (IMSUTH), Orlu, St. John Clinic/Medical Diagnostic Laboratories, Avigram Medical Diagnostic Laboratories, and Qualitech Medical Diagnostic Laboratories. These centers are located in Owerri, Imo State, Nigeria. The Institutional Review Board of the Department of Biochemistry, Imo State University, Owerri, Nigeria, granted approval for this study and all volunteers involved signed an informed consent form. This study was in accordance with the ethical principles that have their origins in the Declaration of Helsinki.

The erythrocytes were washed by centrifugation methods as described by Tsakiris *et al*.[37] Within 2 h of collection of blood samples, portions of 1.0 mL of the samples were introduced into centrifuge test tubes containing 3.0 mL of buffer solution pH = 7.4: 250 mM tris (hydroxyl methyl) amino ethane-HCl (Tris-HCl)/140 mM NaCl/1.0 mM MgCl_2_/10 mM glucose). The erythrocytes were separated from plasma by centrifugation at 1200g for 10 min, washed 3 times by the same centrifugation method with the buffer solution. The erythrocytes were finally resuspended in 1.0 mL of this buffer and stored at 4°C. The washed erythrocytes were lysed by freezing/thawing as described by Galbraith and Watts,[38] and Kamber *et al*.[39] The erythrocyte hemolysate was used for polymerization analyses.

### Polymerization studies

Sodium metabisulfite {Na_2_ S_2_ O_5_; (BDH, UK)}-induced polymerization of HbS molecules was ascertained as described previously by Iwu *et al*[40] with minor modification according to Chikezie *et al*.[41] The underlying principle is that HbS molecules undergo gelation when deprived of oxygen; Na_2_S_2_O_3_ was used as a reductant. The level of polymerization was monitored by recording increasing absorbance of the assay mixture with time. A 0.1 mL of HbS hemolysate was introduced into a test tube, followed by 0.5 mL of phosphate-buffered saline solution (PBS, 9 g NaCl, 1.71 g Na_2_HPO_4_.2H_2_O, 2.43 g NaH_2_PO_4_.2H_2_PO_4_.2H_2_O per liter of distilled water, pH = 7.4) and 1.0 mL of distilled water. The mixture was transferred into a cuvette and 3.4 mL of 2 g% aqueous solution of Na_2_S_2_O_5_ was added. The absorbance of the assay mixture was recorded with a spectrophotometer (SPECTRONIC 20, Labtech—Digital Blood Analyzer^®^) at every 30 s for 180 s at λ_max_ = 700 nm (control sample). This procedure was repeated by substituting the distilled water with 1.0 mL of corresponding four increasing concentrations of the separate extracts (test sample).

$$\mathrm{Percentage}\;\mathrm{polymerization}\;\mathrm{was}\;\mathrm{calculated}\;\mathrm{according}\;\mathrm{to}\;\mathrm{Chikezie}\;et\;al.\left(2010\right)=\frac{At/c\;\times \;100}{Ac180th\sec}$$

Where

At/c =Absorbance of test/control sample at time = *t* s.

Ac_180th s_ = Absorbance of control sample at the 180th s.

### Statistical analyses

The results were expressed in terms of arithmetic means (X) ± standard deviation (SD). The statistical significance of the difference between the means was evaluated by Student’s *t* test.[42]

## RESULTS

**Figure 1:**
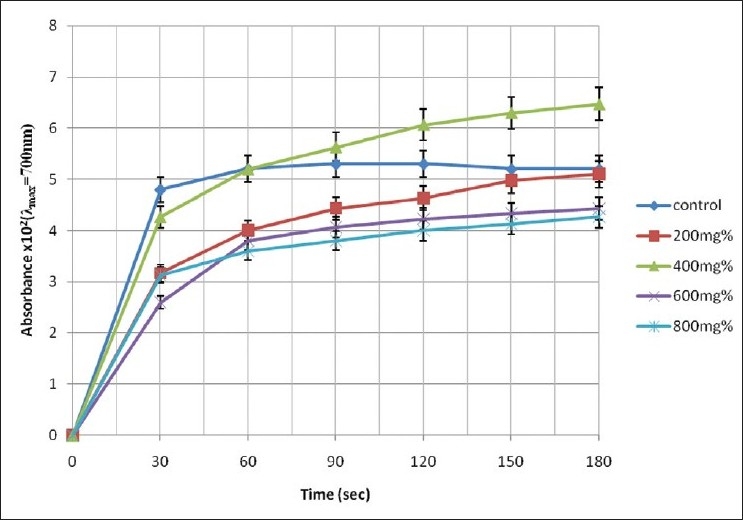
Change in absorbance of erythrocyte haemolysate of HbSS genotype in the presence of aqueous extract of *A. occidentale*

**Figure 2:**
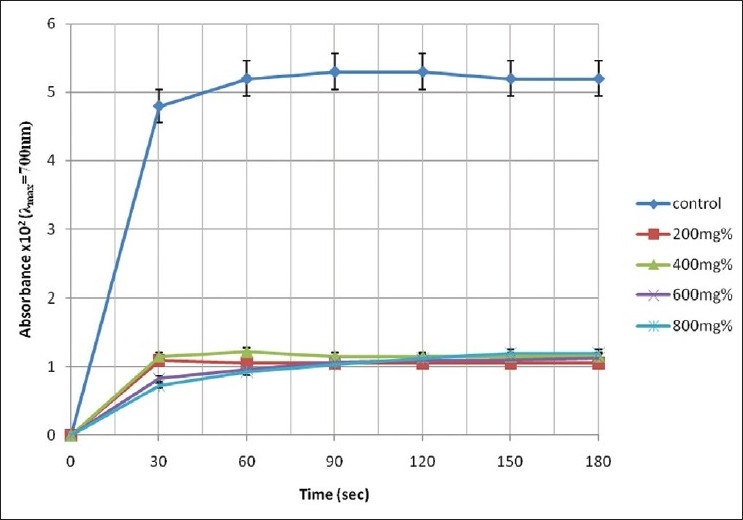
Change in absorbance of erythrocyte haemolysate of HbSS genotype in the presence of aqueous extract of *P. guajava*

**Figure 3:**
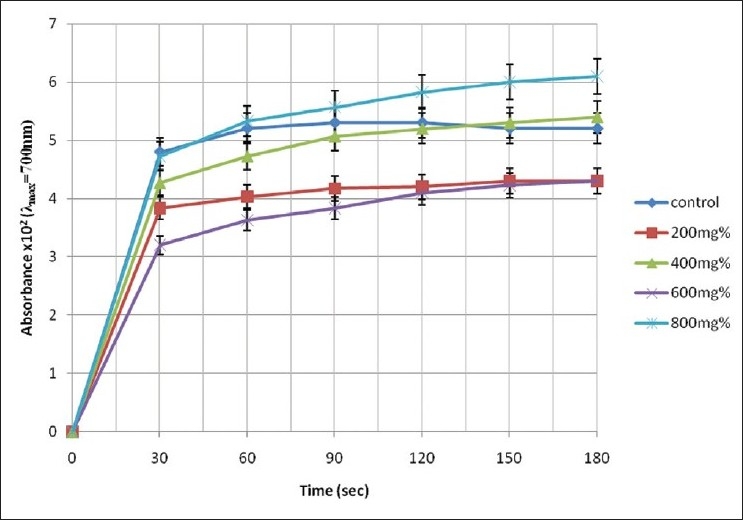
Change in absorbance of erythrocyte haemolysate of HbSS genotype in the presence of aqueous extract of *T. catappa*

The pattern of increase in absorbance of the assay mixture with experimental time is illustrated in Figures 1–3.

A cursory look at Figures 1–3 shows that the rate of increase in absorbance was more rapid within the time range of 0 < *t* < 30 s than subsequent time intervals. The control sample gave absorbance of 0.052 ± 0.05 units at *t* = 180 s representing 100% polymerization of deoxyHbS molecules. However, the test sample containing 400 mg% of *A. occidentale* gave a maximum absorbance of 0.0647 ± 0.004 units at the 180th s (polymerization = 126.86%). Furthermore, Figure 2 shows that the test sample containing 800 mg% of *P. guajava* exhibited the lowest absorbance value of 0/0073 ± 0.01 units at *t* = 30 s corresponding to 14.31 ± 2.11% polymerization [Table 1].

The levels of polymerization of deoxyHbS at specific time intervals compared with the control sample at the 180th s are presented in Table 1. In the control sample, although the levels of polymerization of deoxyHbS molecules were higher at *t* = 90, 120, and 150 s, they were not significantly different when compared with the value at *t* = 180 s (*P* < 0.05). Addition of 200, 600, and 800 mg% aqueous extracts of *A. occidentale* to the assay mixture caused reduction of deoxyHbS polymerization within the experimental period (*t* = 0–180 s). However, the capacity of the three mentioned concentrations of *A. occidentale* to inhibit polymerization of deoxyHbS molecules diminished as the experimental time progressed. Specifically, Table 2 shows that in the presence of extract concentrations of 200, 600, and 800 mg%, the capacity of *A. occidentale* to inhibit deoxyHbS polymerization fell within the ranges of 33.96%-1.92%, 45.83%-14.81%, and 34.79%-17.88%, respectively. Extract concentration of 400 mg% of *A. occidentale* caused reduction in polymerization of deoxyHbS molecules at *t* = 30 s; representing 11.04% ± 1.43% inhibition [Table 2]. Paradoxically, further increases in incubation time engendered activation of deoxyHbS polymerization above the control/reference values (*t* = 180 s; 100% polymerization; Table 2). Furthermore, Table 2 shows at every time interval of 30 s, within the values of *t* > 60 s, the levels of activation gave 6.23% ± 1.34%, 14.53% ± 1.67%, 21.15% ± 1.89%, and 24.42% ± 1.09%.

**Table 1:** Comparative levels of polymerization of deoxyHbS (*t* = 180th s) in the presence of aqueous extracts of *Anacardium occidentale, Psidium guajava,* and *Terminalia catappa*

Time (s)	Polymerization (%)						
	0	30	60	90	120	150	180
Control (n = 9)	0.00 ± 0.00	93.53 ± 1.06	100.00 ± 1.14	101.96 ± 1.13	101.96 ± 2.14	101.37 ± 1.02	100.00 ± 0.65
[*A. occidentale*]; n = 9						
200 mg%	0.00 ± 0.00	62.16 ± 1.03	78.43 ± 1.02	86.86 ± 1.13	90.78 ± 0.95	97.45 ± 0.99	100.00 ± 1.33
400 mg%	0.00 ± 0.00	83.73 ± 0.97	101.96 ± 1.00	110.39 ± 0.95	119.65 ± 1.07	123.53 ± 0.56	126.86 ± 1.78
600 mg%	0.00 ± 0.00	50.98 ± 1.06	74.51 ± 0.98	79.98 ± 0.99	82.94 ± 0.56	84.90 ± 1.22	86.86 ± 1.11
800 mg%	0.00 ± 0.00	61.37 ± 0.96	70.59 ± 1.00	74.51 ± 1.66	78.43 ± 1.88	80.98 ± 0.96	83.73 ± 1.33
[*P. guajava*]; n = 9						
200 mg%	0.00 ± 0.00	21.37 ± 1.34	20.59 ± 2.32	20.59 ± 2.11	20.61 ± 1.88	20.62 ± 2.00	20.63 ± 2.22
400 mg%	0.00 ± 0.00	22.55 ± 0.77	23.92 ± 0.99	22.55 ± 1.23	22.56 ± 1.34	22.57 ± 1.77	22.55 ± 1.00
600 mg%	0.00 ± 0.00	16.27 ± 0.88	19.02 ± 1.54	20.98 ± 1.43	21.57 ± 0.97	21.76 ± 0.98	22.16 ± 1.34
800 mg%	0.00 ± 0.00	14.31 ± 2.11	18.24 ± 1.78	20.20 ± 1.56	22.16 ± 2.00	23.53 ± 1.32	23.53 ± 1.43
[*T. catappa*]; n = 9						
200 mg%	0.00 ± 0.00	75.10 ± 1.34	79.02 ± 2.34	81.76 ± 2.43	82.35 ± 1.09	84.31 ± 1.22	84.31 ± 1.89
400 mg%	0.00 ± 0.00	83.73 ± 1.43	92.75 ± 1.54	94.41 ± 1.43	101.96 ± 1.32	103.92 ± 0.97	105.88 ± 0.76
600 mg%	0.00 ± 0.00	62.75 ± 1.90	71.18 ± 1.43	75.10 ± 1.09	80.39 ± 0.98	82.94 ± 0.89	84.31 ± 1.00
800 mg%	0.00 ± 0.00	92.75 ± 1.23	104.51 ± 0.85	109.22 ± 1.33	114.31 ± 1.11	117.64 ± 1.37	119.61 ± 1.20

The results are means (*X*) ± SD of nine (n = 9) determinations

**Table 2:** Relative levels of inhibition/activation of deoxyHbS polymerization (*t* = 30 s intervals) in the presence of aqueous extracts of *Anacardium occidentale, Psidium guajava*, and *Terminalia catappa*

Time (s)	Polymerization inhibition/activation (%)					
	30	60	90	120	150	180
[*A. occidentale*]; n = 9						
200 mg%	33.96 ± 1.54	23.08 ± 1.09	16.42 ± 0.98	12.64 ± 1.34	4.42 ± 1.11	1.92 ± 1.87
400 mg%	11.04 ± 1.43	0.01 ± 0.11	6.23* ± 1.34	14.53* ± 1.67	21.15* ± 1.89	24.42* ± 1.09
600 mg%	45.83 ± 1.34	26.92 ± 1.76	23.21 ± 1.09	20.19 ± 1.12	16.73 ± 1.66	14.81 ± 2.09
800 mg%	34.79 ± 1.32	30.77 ± 0.99	28.30 ± 0.67	24.53 ± 0.89	20.58 ± 1.67	17.88 ± 1.77
[*P. guajava*]; n = 9						
200 mg%	77.29 ± 1.01	79.81 ± 1.34	80.19 ± 1.78	80.17 ± 1.99	79.78 ± 1.00	79.77 ± 1.37
400 mg%	76.04 ± 1.43	76.54 ± 1.32	78.30 ± 1.11	78.29 ± 1.09	77.86 ± 1.28	77.88 ± 0.99
600 mg%	82.71 ± 1.00	81.35 ± 1.95	79.81 ± 1.45	79.25 ± 0.90	78.65 ± 0.69	78.27 ± 1.56
800 mg%	84.79 ± 1.99	82.12 ± 1.98	80.57 ± 0.67	78.68 ± 1.07	76.92 ± 0.97	76.92 ± 1.43
[*T. catappa*]; n = 9						
200 mg%	20.21 ± 1.77	22.50 ± 1.27	21.32 ± 1.34	20.75 ± 2.04	17.31 ± 1.09	17.31 ± 1.04
400 mg%	11.04 ± 1.54	9.04 ± 1.00	4.34 ± 1.71	1.89 ± 1.90	1.89* ± 1.00	3.85 ± 1.43
600 mg%	37.47 ± 1.48	30.19 ± 1.00	27.74 ± 1.07	22.64 ± 1.45	18.65 ± 1.04	17.31 ± 1.07
800 mg%	1.46 ± 1.66	2.50* ± 1.93	5.09* ± 1.96	10.00* ± 0.99	15.38* ± 1.33	17.31* ± 0.97

The results are means (*X*) ± S.D of nine (n = 9) determinations

* Activation (%)

The four concentrations of *P. guajava* reduced polymerization of deoxyHbS molecules throughout the experimental time [Table 1]. Specifically, aqueous concentration of 800 mg% caused maximum inhibition of 84.79% at the 30th s [Table 2], which corresponded to 14.31% polymerization compared with the control sample at *t* = 180 s [Table 1]. Generally, between *t* = 30-180 s, whereas the diminishing capacity of *P. guajava* extract to inhibit polymerization of deoxyHbS molecules were as follows: 600 mg% (4.44%) and 800 mg% (7.87%), inhibition was sustained and increased by 200 mg% (2.48%) and 400 mg% (1.84%).

Although 800 mg% extract concentration of *T. catappa* hindered polymerization of deoxyHbS by 1.46% at t = 30 s, further increases in experimental time (t > 60 s), engendered activation of the polymerization process. The levels of polymerization were 2.50%, 5.09%, 10.00%, 15.38%, and 17.31% greater than the control sample at corresponding time intervals of 30 s [Table 2]. Also, between the 150th and 180th s, extract concentration of 400 mg% caused activation of polymerization of deoxyHbS molecules by 1.89% and 3.85%, respectively [Table 2]. The maximum capacity of aqueous extract of *T. catappa* to inhibit polymerization of deoxyHbS molecules occurred at t = 30 s, in the presence of extract concentration of 600 mg%. However, the level of inhibition attenuated as experimental time progressed.

## DISCUSSION

Noteworthy, polymerization of deoxyHbS is dependent on physiologic concentration of 2,3-DPG.[43] However, according to Ferrone *et al*,[44] 2,3-DPG contributes only a minor effect in predicting intracellular polymer formation. Therefore, in this study, the interplay of physiologic concentration of 2,3-DPG on HbS polymerization was neglected.

An overview of the present study showed increasing level of polymerization of deoxyHbS molecules with time that is consistent with the pattern described elsewhere.[41,45,46] *In vitro* deoxygenation of hemolysate HbS molecules by sodium metabisulfite caused progressive aggregation and polymerization of the individual hemoglobin molecules. The process of gelation (polymerization) of hemoglobin molecules resulted in increasing absorbance of the assay solution with time.

Although after 60 s, extract concentrations of 400 mg% of *A. occidentale* and 800 mg% of *T. catappa* activated polymerization of deoxyHbS molecules, the present study showed that aqueous extracts of the three medicinal plants (*A. occidentale*, *P. guajava*, and *T. catappa*) exhibited variable capacity to hinder polymerization of deoxyHbS molecules. In conformity with earlier reports of Oyewole *et al*,[47] a measure of inhibition of deoxyHbS polymerization by these extracts was found to be dose and time dependent. Previous reports have proposed the use of herbal preparations as candidate for management of sickle cell disease.[46–52] Their proposals were drawn from the fact that these plant extracts, from *in vitro* studies, exhibited antisickling/polymerization property. The findings of this research are comparable to those previous reports. Research findings have established that the capability of a biomolecule to impede *in vitro* polymerization depends on one or combinations of the following options.

- The tendency and efficiency to bind to the complimentary contact region/site of deoxyHbS monomers.[34–36]
- Modification of amino acid residues that contribute to the three-dimensional structures of HbS contact region and other critical sites.[32,33,47]
- Stabilization of the R (relaxed) state of HbS molecule.[32,47,53,54]

The diminishing capacity of the three plant extracts to inhibit polymerization of deoxyHbS molecule with progression of experimental time suggest that the constituents of the extracts did not covalently modify the amino acid residues unlike other reported compounds.[25,31–36] Rather, the antipolymerization principles of the plant extracts may have formed a relatively weaker hydrophobic interactions with the contact regions of HbS molecules that temporarily reduced polymerization of HbS monomers. Furthermore, the protein/ligand associations may have transiently stabilized the R-state conformation, but were subsequently displaced by more thermodynamically favorable interactions that engendered and promoted hemoglobin polymerization. Therefore, the capacity of the three extracts to inhibit HbS polymerization was not sustained with the progress of experimental time.
